# Treatment of membranous nephropathy: Perspectives on current and future therapies

**DOI:** 10.3389/fneph.2023.1110355

**Published:** 2023-01-27

**Authors:** Monarch Shah, Andrew DeLaat, Corey Cavanaugh

**Affiliations:** ^1^ Division of Nephrology, University of Virginia, Charlottesville, VA, United States; ^2^ Liberty University College of Osteopathic Medicine, Lynchburg, VA, United States

**Keywords:** membranous nephropathy, PLA2R, B-lymphocytes, cyclophosphamide, rituximab, complement, CD19, CD20

## Abstract

Primary membranous nephropathy remains one of the most frequent causes of nephrotic syndrome in adults. It is an autoimmune disorder in which auto-antibodies target antigens at the podocytes cell membrane–basement membrane interface. Our understanding of membranous nephropathy has expanded dramatically as of late. After the initial discovery of the phospholipase A2 receptor auto-antibody in 2009, eight more antigens have been discovered. These discoveries have led to refinement in our understanding of the pathogenesis, diagnosis, and natural history of primary membranous nephropathy. Now, many experts advocate for redefining primary membranous nephropathy based on antigen, potentially shedding the primary and secondary nomenclature. Recently, therapies for primary membranous have also expanded. Immunosuppressive therapies like cyclophosphamide and rituximab, which primarily target B-cells, remain the cornerstone of therapy. However, there is still significant room for improvement, as many as 30-40% do not respond to this therapy according to recent trials. Additionally, drugs targeting complement, and other novel therapies are also under investigation. In this review we will discuss the available therapies for primary membranous nephropathy in light of recent clinic trials like GEMRITUX, MENTOR, RI-CYCLO, and STARMEN, as well as management strategies. While the last 10 years have seen a boom in our mechanistic understanding of this ever-diversifying disease, we are likely to see a similar boom in the therapeutic options in the years to come.

## Introduction

Membranous nephropathy (MN) is a common cause of nephrotic syndrome, particularly in white adults of middle age. Although approximately 30% undergo spontaneous remission, an equal number of patients experience renal failure at 15 year follow up who do not respond to immunotherapy (1). Renal survival in the study by Troyanov et al. was 100% in patients with complete remission and 70% for partial remission. Therefore, theoretically with appropriate treatment and appropriate timing, the risk of renal failure can all but be eliminated.

MN has undergone extensive change in the last 10 years. The number of target antigens discovered has exploded. Now totaling 10 separate antigens at the time of this writing, the vast majority of patients with MN now have an identifiable antigen. The clinicopathologic findings appear distinctive for the antigens (2). For example, semaphorin 3B-associated MN is seen typically in pediatrics, while protocadherin 7 – associated MN has minimal complement deposition on histopathologic examination (3, 4). While a deeper understanding of pathogenesis in MN is evident, applying targeted treatment regimens for the antigens beyond PLA2R remains an open question. In this review will discuss optimal care for membranous nephropathy, and focus on recent clinical trial data and B cell depleting therapy.

## Non immunosuppressive management

Supportive care in MN often involves an initial attempt to control proteinuria and blood pressure with angiotensin-converting enzyme (ACE) inhibitors or angiotensin receptor blockers (ARB) and cholesterol control with statins, given the risk of cardiovascular events in MN. Recent large epidemiologic studies have revealed MN carries significant risk of cardiovascular events (5, 6). Utilizing electronic health records of a population of 907 patients with glomerular disease, adults with nephrotic syndrome from primary glomerulopathy had over a 2.5-fold increased adjusted rate of incident acute coronary disease compared to matched controls (aHR, 2.58; 95% CI, 1.89 to 3.52) (6). Similar to these findings, a study by Canney et al. in 2022 noted a 3 fold increase in rate of heart failure (aHR, 3.01; 95% CI, 2.16 to 4.19) and a nearly two-fold increased adjusted rate of ischemic stroke (aHR, 1.80; 95% CI, 1.06 to 3.05) in a multivariable analysis (5).

According to the KDIGO guidelines, patients with MN and proteinuria should receive conservative treatment such as diuretics, ACE/ARBs, sodium restriction with the goal to decrease edema and prevent cardiovascular events (7). Renin-angiotensin system (RAS) inhibition has long proven beneficial for proteinuric kidney disease and was an inclusion criteria for many recent controlled trials, however the evidence of its benefit in nephrotic syndrome is somewhat conflicting. Although RAS inhibition with ACE inhibitor or ARB can reduce glomerular intracapillary pressure and improve size selectivity, most of these studies evaluating RAS inhibitor use in nephrotic syndrome were small and heterogeneous (8–11). Praga et al. showed in a small prospective study that there was no reduction in proteinuria in patients with > 5g/d proteinuria and hypoalbuminemia when treated with ACE inhibitors (8). A multicenter retrospective study (N=328) from the Spanish group for the study of glomerular disease (GLOSEN) with idiopathic MN revealed patients treated with ACE inhibitors or ARBs had significantly higher rate of spontaneous remission (79.8% versus 60.7%, P = 0.009) (12). However, this was restricted to patients with <8g/day proteinuria at baseline, thus a higher baseline rate of spontaneous remission is expected. The results of DAPA-CKD, and the recently published EMPA-KIDNEY compared SGLT2 inhibitors to placebo in non-diabetic, CKD patients. These large trials (>10,000 patients combined) proved there was a reduced risk of progressive CKD in non-diabetic kidney disease including glomerular diseases, however a minority of patients (43 in DAPA-CKD) included had MN (13, 14). Yet it is sure to be argued the precedence set forth by ACE/ARB therapy in glomerular disease should be extended to SGLT2i in light of these trials, for its well proven benefits in CKD, and heart failure outcomes, which occur at a high rate in glomerular disease and MN.

## Perspectives on current immunotherapies in primary membranous nephropathy

Targeting B cells, with alkylating agents (chlorambucil, cyclophosphamide) or CD20 targeting antibodies has been an effective therapy in MN for decades. The use of the anti-CD20 monoclonal antibodies like Rituximab (RTX) has now become commonplace for treatment of MN and is favored by many nephrologists for its side effect profile, ease of use, and limited monitoring. By inducing B cell depletion with a CD20 targeting monoclonal antibody and ultimately the autoantibody it will produce, the injury to the podocytes is limited. Additionally, calcineurin inhibitors (CNI) like tacrolimus and cyclosporine, have long been established as a therapy for MN. Although less effective in auto-antibody depletion, they have powerful anti-proteinuric effects by stabilizing the actin cytoskeleton of podocytes *via* its preservation of synaptopodin (15, 16). Supporting the targeted effects of these drugs, Haddad et al. showed in a cell culture model of PLA2R-overexpressing podocytes, sera from anti-PLA2R positive MN patients induced proteolysis of synaptopodin and NEPH1, two key cytoskeletal proteins of the podocyte (17). There is now robust randomized control data clarifying best use of each agent, which will be discussed below. The KDIGO 2021 treatment guidelines for MN have been updated with the addition of rituximab as seen in **Table 1**, however there are a number of unanswered questions that remain, thus an opportunity for innovation and improved outcomes in MN.

**Table 1:** Risk assessment and treatment of membranous nephropathy per KDIGO Clinical Practice Guidelines (7).

RISK	Low risk Normal eGFR, <3.5g proteinuria & serum album >3 g/l Or Normal eGFR, proteinuria <3.5g/d or a decrease >50% after 6 months conservative therapy ACEi/ARB	Moderate risk Normal eGFR, proteinuria >3.5g/d and no decrease >50% after 6 months conservative therapy ACEI/ARB AND Not fulfilling high-risk criteria	High risk egfr < 60 ml/min 1.73m2 and/or >8 g proteinuria for >6 months OR Normal eGFR, proteinuria >3.5g/d and no decrease >50% after 6 months conservative therapy ACEi/ARB With at least one of the following: Serum albumin <25g/l PLA2R > 50 RU Serum Albumin <2.5g urinary α1M >40 μg/min; urinary β2M >1 μg/min; urinary IgG >250 mg/day Selectivity in > 0.20	Very high risk Life threatening nephrotic syndrome or Rapid decline in Kidney function
Treatment	Monitor ACEI/ARB	Monitor rituximab* Calcineurin inhibitor + glucocorticoids	Rituximab* cyclophosphamide + glucocorticoids Calcineurin inhibitor + rituximab	Cyclophosphamide + Glucocorticoids

eGFR, estimated glomerular filtration rate; PLA2R Ab, M-type phospholipase A2 receptor antibody.

## Recent clinical trials in primary membranous nephropathy

The GEMRITUX and MENTOR trials were the initial controlled trials that solidified rituximab’s role in MN. In 2017 GEMRITUX compared rituximab given as two infusions of 375mg/m^2^ plus non-immunosuppressive anti-proteinuric treatment (NIAT) compared to NIAT alone (18). GEMRITUX did not reach its primary end point of complete and partial proteinuria remission at 6 months, as no statistically significant difference was found between RTX and NIAT. However, in the post-trial observational period proteinuria remission rate was substantially higher in the NIAT-rituximab group than in the NIAT alone group (64.9% versus 37.5%) (18). In line with trials that would follow, 36.1% in the NIAT-rituximab group did not respond after a 17-month median duration follow up. In 2019, the MENTOR trial compared cyclosporine to rituximab for MN (19). Rituximab was given as two infusions (1gram, 2 weeks apart with repeat dosing at 6 months if proteinuria reduction ≥ 25% at 6 months without complete remission) and was found to be non-inferior to cyclosporine at 1 year in high-risk MN patients. 39 of 65 patients (60%) in the rituximab group and 34 of 65 (52%) in the cyclosporine group had a complete or partial remission (risk difference, 8%; 95% confidence interval [CI], −9 to 25; P=0.004 for noninferiority). After 2 years rituximab proved superior, 39 patients (60%) in the rituximab group compared to 13 (20%) in the cyclosporine group achieving complete or partial remission (risk difference, 40%; 95% CI, 25 to 55; P< 0.001 for noninferiority and superiority). The difference in outcomes was due to a high relapse rate in the cyclosporine group of 52.9% after 2 years, compared to rituximab at just 5.1%. Serious adverse events (SAE) also favored rituximab at 17%, compared to cyclosporine with 31%. Additionally, 8/65 (12%) in the cyclosporine group saw a ≥50% decline in creatinine clearance compared to 1/65 (1.5%) in the rituximab group at 1 year. Although most patients saw either complete or partial response in the rituximab group, this response was not immediate. By 6 months, zero patients achieved complete remission, while at 12 months, only 9 of the 65 patients (13.8%) saw complete remission in the rituximab group. Similarly, only 1.5% and 4.6% had complete remission at 6 and 12 months respectively for cyclosporine. Most patients in the rituximab group received repeat dosing at 6 months, per study protocol, unlike GEMRITUX. After 2 years, up to 40% of patients in the rituximab arm did not respond. The efficacy of rituximab over cyclosporine is likely explained by its prolonged impact on immunological remission, thus preventing immediate relapse after drug withdrawal as seen in cyclosporine. Although not a primary outcome measure, patients who achieved complete or partial remission with rituximab had a 24.4% (27.1-17.7) greater reduction of anti-PLA2R antibody levels at 6 months, and 21.3% (24.9-11) reduction at 18 months, when comparted to cyclosporine.

Although MENTOR and GEMRITUX proved rituximab’s efficacy in MN, nephrologists were left wondering how the long-proven therapy of alternating cyclophosphamide and glucocorticoids (CYC-GC or modified Ponticelli regimen), would compare to rituximab in a controlled trial. In 2021 two studies were published exploring the use of CYC-GC compared to a rituximab-based regimen. STARMEN compared CYC-GC to a sequential regimen of tacrolimus (TAC) for 6-9 months, with a single 1gram dose of RTX at month 6 (20). The primary endpoint was complete or partial proteinuria remission at 2 years. The hypotheses of the benefit behind sequential tacrolimus and rituximab are numerous. First, early proteinuria reduction and thus symptom relief with tacrolimus and its podocyte stabilizing properties. Secondly, TAC may prolong the half-life of rituximab by its proteinuria control. Third, it would avoid the calcineurin inhibitor withdrawal relapse, *via* overlapping with rituximab. Lastly it could limit T helper cell activity in the humoral response. In STARMEN, 86 patients were randomized to the TAC-RTX and CYC-GC regimens. 36/43 (83.7%) in the CYC-GC group compared to 25/43 patients (58.1%) in TAC-RTX group had a primary outcome of complete or partial remission at 24 months (relative risk [RR] 1.44, 95% confidence interval [CI] 1.08 to 1.92). The occurrence of remission was also faster in the CYC-GC group with 51% achieving complete or partial remission by 3 months compared to 32% in the TAC-RTX group in the per-protocol analysis (RR 1.65, 95% CI 0.96 – 2.85). However, there were more adverse events in the CYC-GC group compared to the TAC-RTX group (239 vs 170 respectively), with 10 serious adverse events in the CYC-GC group compared to 6 in TAC-RTX. 30% of patients in the CYC-GC group experienced leukopenia, but no fatalities were recorded in either group. Also somewhat surprising is the rate of infusion reaction to rituximab was much lower at 4/43 (9%) in STARMEN, compared to other trials in MN, 25% in MENTOR and 24% in the later discussed RI-CYCLO trial. In addition to STARMEN, RI-CYCLO was published the same year (21). A long-awaited pilot study of 74 patients randomized to cyclic regimen of cyclophosphamide and corticosteroids was compared to rituximab infusion (1gram, 2weeks apart) in MN. Although the trial failed to detect a statistically significant difference in primary outcome, *half* as many participants in the rituximab arm (*n*=6; 16%) achieved complete remission at 12 months compared to cyclophosphamide group (*n*=12; 32%) (odds ratio [OR], 0.40; 95% CI, 0.13 to 1.23) with similar rates of adverse events. Unlike the MENTOR trial, rituximab was not re-dosed if there was a poor proteinuric response in Ri-CYCLO. As others have pointed out, this renders the possibility of inadequate dosing of rituximab to explain a lower response rate (22).

As it stands, CNI, rituximab, or CYC-GC are acceptable treatments for moderate to high-risk MN per KDIGO, while CYC-GC alone is indicated for the very high-risk category patient with MN as initial therapy. The decision between the three regimens can be nuanced and involves extensive discussion with the patient. On the one hand cyclosporine was shown to be inferior to rituximab with a similiar risk of infection and it was coupled with a higher rate of hyperkalemia, creatinine elevation, hypertension and the need for drug level monitoring. On the other hand, cyclosporine is often easier to access and cheaper for patients, while rituximab requires minimal monitoring, but it is expensive and frequently denied coverage by insurance companies. Despite decades of use and now several controlled trials, there are still uncertainties regarding B cell depleting therapies in MN. Questions regarding dosing of rituximab, duration, and resistance to rituximab are still not completely answered as per **Table 2**. Importantly as these trials show, 20 to 40% do not respond to rituximab, and a significant number can progress to end stage kidney disease. This leaves the nephrologist often turning to CYC-GC in situations of ‘rituximab resistant’ cases, or if in the very high-risk category, starting with CYC-GC as initial therapy. In the following section, we will explore these open questions regarding B cell targeting therapy in MN and alternatives to traditional CYC-GC and rituximab regimens.

**Table 2:** Recent Clinical Trials in PMN.

Study	N	Proteinuria	GFR	Intervention	Complete or partial remission (Complete remission)	References
2017 GEMRITUX	77	7.3 g/24 hr	>60	Two doses of 375 mg/m^2^ rituximab vs. conservative therapy (NIAT)	Rituximab + NIAT: 65% at 17 months (CR 19%) NIAT: 34% at 17 months (CR 3%)	(18)
2019 MENTOR	130	8.9 g/24 hr	>40	Rituximab: two infusions, 1000 mg each, administered 14 days apart; repeated at 6 months if partial response. VS Cyclosporine	Rituximab: 62% at 18 months (CR 28%) Cyclosporine 33% (CR 2%)	(19)
2021 RI-CYCLO	74	6 g/24 hr	≥30	Rituximab: two infusions, 1000 mg each, administered 14 days apart VS. 6-month cyclic regimen with corticosteroids alternated with cyclophosphamide every other month	Rituximab: 66% at 18 months (CR 31%) CYC-GC: 79% at 18 months (CR 21%)	(21)
2021 STARMEN	86	7.4 g/24 hr	≥45	6-month cyclic regimen with corticosteroids alternated with cyclophosphamide every other month vs. Tacrolimus-Rituximab (Tacrolimus 0.05mg/kg/day first 6 months, followed by Rituximab 1 gram).	CYC-GC: 84% at 18 months (CR 44%) Tacrolimus-rituximab: 53% at 18 months (CR 16%)	(20)

NIAT, non-immunosuppressive anti-proteinuric treatment; CYC-GC, cyclophosphamide - glucocorticoids.

## Cyclophosphamide and glucocorticoids

Cyclic use of cyclophosphamide and corticosteroids have proven to be a highly effective therapy for MN for many years and is the only therapy with proven improved kidney survival, and thus remains the only therapy recommended for very high-risk patients per KDIGO (7, 23, 24). In two controlled trials of CYC-GC with long term follow of 6 and 10 years up by Jha and Ramachandran, the combined complete and partial remission was 72 and 86% respectively (24, 25). Additionally, in a study by Van de Logt et al, a cohort of patients with positive anti-PLA2R antibody MN, cyclophosphamide had a similar effect on PLA2R antibody level disappearance regardless of level after 6 months (26). However, those treated with rituximab saw a substantial drop off in the disappearance of PLA2R antibodies at 6 months in the group with PLA2R levels above 152 RU/ml, arguing rituximab is less effective than cyclophosphamide in inducing immunologic remission in patients with MN when antibody levels are high. Although highly efficacious, there is hesitation to use cyclophosphamide. It is associated with several significant toxicities including infection, myelosuppression, liver dysfunction, infertility and malignancy. Modern studies such as RI-CYCLO and STARMEN have shown an incidence of major infection of 8 and 12% respectively (20, 21). The risk of malignancy is a major concern for clinicians when using CYC, which is largely dependent on cumulative dose. The risk of bladder cancers and leukemia increases after 36g. However, with typical doses of CYC in membranous nephropathy being much lower (9-13g), only a higher risk of non-melanoma skin cancer was observed among patients with granulomatosis polyangiitis (27). While both the efficacy and toxicity of CYC in MN has long been evident, there is likely substantial added risk imposed by glucocorticoids in the cyclic CYC-GC regimen. The KDIGO guidelines have long held that monotherapy with glucocorticoids is not beneficial in MN, it is believed cyclophosphamide and glucocorticoids are synergistic although controlled trials supporting this notion are lacking in MN (7). There is a trend to use steroid sparing regimens in other glomerular disease (Lupus, MCD, ANCA), as high-dose corticosteroids, and pulse methylprednisolone are major contributors to the risk of serious infection (28, 29). Coupled with a risk of high dose steroids and complex execution of the modified Ponticelli regimen for MN, a number of variations have been tried (**Table 3**). Zonozi et al. have reported results in using combination CYC, rapid prednisone taper, and 2 years of rituximab (Cortazar Regimen in **Table 3**) with an impressive 100% response rate in their single center retrospective study (30). This was achieved with a low dose prednisone protocol (2.63 grams) and CYC (5.95g) thus adverse events were low at 7.9 SAEs per 100 patient years, whereas MENTOR saw 10 and 17 SAE per 100 patients in the rituximab and cyclosporine arms respectively. Surprisingly, MN has not seen the transition to intravenous CYC as the recommended regimens have done for lupus nephritis, ANCA vasculitis and others. This is due to simply a dearth of studies supporting this, but there is no apparent reason to doubt its efficacy and safety when delivered as an IV regimen. Recently Luzardo et al. reported on their experience in replacement of oral cyclophosphamide with a single intravenous pulse on months 2, 4, and 6 as per the modified Ponticelli regimen (31). They found this regimen was effective and safe with 39 (71%) patients achieved clinical response with complete remission observed in 23 patients (42%) and partial remission in 16 (29%) at 24 months. None of the 55 patients received >3g cumulative dose of cyclophosphamide, nor required transfusion or had evidence of myelosuppression, with only one developing infection (pneumonia) requiring hospitalization. While IV cyclophosphamide for MN or combining with an extended duration rituximab is far from routine, in our experience there is no advantage no advantage in efficacy or safety compared with alternating CYC and CS. We prescribe them concurrently as other centers have done, giving oral CYC for 3 consecutive months with an abbreviated (6 to 8 weeks) prednisone taper, holding the pulse of methylprednisolone, with favorable results. While new therapies are on the horizon, many patients are sure to have limited access to them. It is worth investigating new methods with existing therapies to maximize efficacy, while limiting risk and cost.

**Table 3:** Summary of select cyclophosphamide-based regimens in membranous nephropathy.

Cyclophosphamide based regimens	Drugs, Dosage	Cumulative Dose for 60 kg patient	References
Modified Ponticelli	Months 1,3,5: 1 gram methylprednisolone (MP) IV days 1-3, Followed by oral prednisolone 0.5mg/kg x27 days Months 2,4,6: 2-2.5mg/kg oral cyclophosphamide daily	2.43g Oral prednisone + 9g IV MP Total GC dose =11.43g Total CYC dose = 10.8 – 13.5 g	(21, 23)
Cortazar regimen	Prednisone initiated at 60 mg daily, tapered to 15 mg daily by 4 weeks, and then slowly tapered to complete a 6 month Cyclophosphamide oral 2.5 mg/kg daily for 1 week, then 1.5 mg/kg for daily x 7 weeks Rituximab: 1g IV x2 separated by 2 weeks. 1g IV every 4 months x 2 years	Total oral prednisone dose=2.63g Total CYC dose = 5.46g	(30)
IV Alternating CYC – GC	Months 1,3,5: 1 gram methylprednisolone (MP) IV days 1-3, Followed by oral prednisolone 0.5mg/kg x27 days Months 2,4,6: 15 mg/kg IV single dose on day 1	2.43g Oral prednisone + 9g IV MP Total GC dose =11.43g Total CYC dose = 2.7g	(31)
Dutch Protocol	1 gram methylprednisolone (MP) IV days 1-3 months 1,3,5, plus oral prednisolone 0.5mg/kg x 6 months and tapered 1.5-2.0 mg/kg oral cyclophosphamide daily x 12 months.	5.4g oral prednisone (first 6 months) 9g IV MP Total GC dose = 14.4g Total CYC dose = 32 – 43.8g	(32)
Compressed & concurrent CYC – GC protocol	Months 1-3: Prednisone 0.5mg tapered over 6-8 weeks, Cyclophosphamide oral 1.5-2mg/kg daily	~80-100mg oral prednisone Total CYC dose = 8.1-10.8 g	

## CD20 targeting therapy

Despite differences in trial design, patient population, and other baseline characteristics, at 18 months of MENTOR, GEMRITUX, Ri-CYCLO and STARMEN only 53%- 66% of patients had a complete or partial response to rituximab (18–21). A similar rate of remission of 67% was found in a recent meta-analysis of 21 studies of rituximab in MN (33).The combination of high cost and varied response has led to an interest in tailoring the dose for optimal response, and to explore mechanisms of resistance (**Table 4**). Common dosing strategies by clinicians follow the MENTOR trial with a dose of 1 g × 2 doses separated by 2 weeks, others choose a four 375-mg/m2 weekly dose regimen. Although doses as low as 100mg, single dose 375m2, and 500mg with repeat dosing have been used successfully in MN, other authors have described a peripheral B-cell monitoring strategy to adjust Rituximab doses, with favorable results (44, 45). There is no clear superior regimen of rituximab in MN, however the search for methods to refine dosing are ongoing and will be discussed below. Patients with more severe nephrotic syndrome have a more rapid elimination of rituximab, as is the case for all IgG when the selectivity index is high in MN (46–49). This has led some to recommend earlier re-dosing (e.g. 3-4 month interval after initial dosing as opposed to 6 months) to potentially account for these losses. Seizt-Polski et al. compared the result of the GEMRITUX cohort, that received 1.4g cumulative dose of rituximab vs the NICE cohort that received 2g (50). They noted the higher rituximab dose protocol of the NICE cohort, which achieved both earlier and more frequent remission, had higher residual rituximab levels at 3 months and lower CD19 counts. However, the primary limitation of this study is the retrospective nature, and that GEMRITUX cohort also had higher proteinuria at baseline, arguing that lower rituximab levels at 3 months could simply mean more rituximab loss in the urine. Indeed, the rate of PLA2R antibody depletion at 3 months was similar between the groups 16/27 (59%) in the NICE cohort vs 14/25 (56%) in the GEMRITUX cohort. In a separate retrospective analysis, an undetectable serum rituximab level at month-3 was an independent risk factor for treatment failure at 6 and 12 months in a cohort of 68 patients with MN (36). Although again the main limitation being the retrospective nature of the study and that patients with <2µ/ml serum rituximab level at 3 months had higher proteinuria than patients with >2µ/ml serum rituximab, representing worse disease. Rituximab level monitoring will require analysis with a controlled trial before this practice can be recommended to guide dosing strategy.

**Table 4:** Potential mechanisms of rituximab resistance and strategies to overcome.

Mechanism	Notes and treatment strategies	References
Internalization of type I anti-CD20 mAbs from the surface *via* FcγRIIb, B cells unable to undergo ADCC	Shown *in vitro* for rheumatoid arthritis, SLE and malignancy. Consider using Type II CD 20 antibody (obinutuzumab) vs Type I (rituximab)	(34, 35)
Neutralizing anti-rituximab antibody formation	Anti-rituximab abs seen in in 23% of patients with MN 6 months after rituximab, associated with faster B cell reconstitution.	(36, 37)
CD 19/20 negative plasma cell production of autoantibody	Plasma cell targeting therapy. Studies are ongoing, limited to case reports	(38, 39)
Tertiary lymphoid structures (TLS) produce autoreactive B cells within kidney	Prolonged b cell depletion, B cell activating factor (BAFF) targeting with belimumab	(40)
Lack of T cell targeting. Loss of rituximab *via* proteinuria	Add on calcineurin inhibitor	(36, 41–43)

## Autoantibody and B cell monitoring in B cell depleting therapy

Observational studies have shown immunologic monitoring with serial anti-PLA2R antibody levels can predict proteinuria changes in MN, months in advance (51–54). Current KDIGO guidelines recommend serial monitoring of PLA2R antibodies at 3-month intervals, and potentially more frequently if in high-risk category (7). In a prospective study by Jatem-Escalante et al, baseline anti-PLA2R antibody < 97.5 carried a sensitivity and specificity of 71% and 81% respectively for the primary endpoint of ≥50% reduction in proteinuria by 6 months (55). The prediction of the primary endpoint could be enhanced, if the baseline PLA2R antibody declined by at least 15% at 3 months on repeat PLA2R antibody level (sens. 93%, spec. 80%). KDIGO guidelines now suggest stopping immunosuppressive therapy when serum PLA2R ab is absent after 6 months. However, none of the controlled trials included PLA2R antibody depletion as an endpoint. 30% of all patients with MN are PLA2R negative, and only 53%- 66% achieve a proteinuria response at 18 months, thus the clinician is forced to monitor response for extended time without a specific and reliable marker to follow. There is early evidence that circulating antibody removal correlated with remission in the more uncommon antigens (NELL-1, and THSD7A) (56, 57). Future methods in disease monitoring are likely to involve use of novel circulating antibodies to target antigens similar to PLA2R (THSD7A, NELL-1 etc.) to guide therapy, however more studies will be needed to ensure validity of this practice.

## B cell monitoring

Some clinicians prefer to monitor peripheral b-cell counts (CD19/CD20) to signal anti-CD20 response. However, studies evaluating CD19 counts as a treatment target have mixed results in several diseases, membranous nephropathy included. Most patients experience rapid peripheral B cell depletion within days of rituximab administration. Studies did not reveal a correlation between CD19/20 count and proteinuric remission, and many patients remain in remission despite B cell repopulation, or achieve a response without depletion (44, 58, 59). Anti-CD20 antibodies like rituximab have little impact on CD20 negative plasma cells, the major antibody secreting cell. Therefore, the goal of anti-CD20 antibodies is to deplete the pool of memory B cells (mBCs) to prevent their conversion into auto-antibody producing plasmablasts. Supporting this concept in MN, Canteralli et al. showed there is a significant association between the percentage of circulating plasma cells, generated from PLA2R-specific memory B cells mBCs, and anti-PLA2R IgG levels in PLA2R positive patients with MN (P < 0.001) (60).

The circulating B cell population may not represent the total B cell population in MN, as interstitial CD20 positive B cells did not reflect the circulating B cell value in lupus (61). CD20 positive B cell infiltrate in the kidney has also been seen as a marker of poor outcomes in SLE, ANCA, and now MN (62, 63). B cell lymphocytes organize with T cells and dendritic cells forming tertiary lymphoid structures (TLS) resembling lymph nodes, but are limited to the kidney interstitium. The presence of TLS has been associated with local autoantibody production in SLE (64). A study by Fleig et al. recently showed evidence of complex B cell structures in renal interstitium of patients with membranous nephropathy, consistent with TLS formation (40). In their analysis of 63 patients after kidney biopsy with membranous nephropathy (35 of which were treated with rituximab), they showed CD20 positive B cell infiltrates in the kidney was associated with worse eGFR, and improvement in kidney function (eGFR) after prolonged B cell depletion of 18 months. The retrospective study is limited by the heterogeneous patient population - a number of which had prior exposure to immunosuppressive therapy (including CNI), and lack of standardized monitoring (pla2r, repeat biopsy). However, it raises the interesting point that while peripheral B cell monitoring may not yield prognostic information, interstitial B cells could be of pathogenic relevance and could be a target for treatment.

## Future therapies in membranous nephropathy

In MN, the number of circulating plasma cells is abnormally high (41, 60). Non-proliferating long-lived plasma cells form the basis for humoral memory and can result in refractory autoimmune disorders by producing significant levels of IgG autoantibodies and alloantibodies (65–68). Auto-reactive plasma cells which lack CD19 and CD20 are resistant to rituximab, and may be a primary cause of rituximab resistance in MN among other potential mechanisms as seen in **Table 4**. Plasma cell targeting therapies like bortezomib, and daratumumab could be considered for the treatment of resistant MN as they have been effective in case studies (38, 39, 69). Other drugs targeting the plasma cell markers CD138, and CD38 like daratumumab, are under investigation for MN. Trials currently exploring the anti-CD38 antibody MOR202 (Felzartamab) in patients for resistant MN, or who have failed CD 20 targeted therapy are eagerly awaited (NCT04893096, NCT04145440).

Second and third generation fully humanized anti-CD20 targeting antibodies like obinutuzumab, ocrelizumab and oftatumumab are often used, but have not been systemically studied in “rituximab resistant” cases of MN, and their proven efficacy is limited to case studies (70–72). Obinutuzumab has shown impressive B cell depletion rates in the phase II NOBILITY trial in SLE with lupus nephritis, with sustained B cell depletion in 32/52(62%) at week 76 (73). On the other hand, a phase III trial evaluating ocrelizumab in lupus nephritis was halted early for high rate of serious infection in the ocrelizumab arm (74). These newer agents will require controlled trials, ideally against existing therapies like rituximab before they can be recommended.

Monoclonal antibody belimumab which was recently approved by the FDA for use in SLE and lupus nephritis, functions by inhibiting BAFF the B-cell activating factor (75). In an open-label, prospective, single-arm research involving 14 patients with PLA2R-positive MN, belimumab reduced proteinuria and circulating PLA2R antibody titers by 86% and 97%, respectively (76). The ongoing investigator lead controlled trial REEBOOT (Belimumab with Rituximab for Primary Membranous Nephropathy) is enrolling patients across North America to evaluate its efficacy in combination with rituximab compared to rituximab alone in PLA2R positive patients (NCT03596385). Study completion is not expected until 2026.

## Complement targeting

As an immune complex disease, complement mediated injury is integral in the pathogenesis of MN, and thus a viable therapeutic target. In PLA2R MN, IgG4 is the predominant immunoglobulin targeting the PLA2R antigen. IgG4 cannot activate complement *via* the traditional route, however it was recently shown hypoglycosylated IgG4 present in the sera of MN patients is capable of complement activation (17). According to recent proteomic analyses of kidney biopsies with membranous nephropathy, complement ranked amongst the most abundant proteins found. Multiple investigators have found evidence of the classical, lectin, and alternative pathways undergoing activation, with C3 and C4 being the most abundant (77, 78). A new avenue of complement targeting therapy in MN is being studied. In a mouse model of MN, the use of the small-molecule factor B inhibitor iptacopan, (LNP023) an inhibitor of C3 convertase improved proteinuria and halted disease progression (79). Glomerulopathy and tubular damage improved on histopathologic examination with an absence of C3 staining, indicating that the alternative complement pathway had been successfully blocked and prevented complemented mediated injury. Effects on proteinuria and histology were shown independently of iptacopan use as a preventative measure or treatment of resultant proteinuria. This drug will be tested against rituximab in an ongoing phase II trial (NCT04154787). Targeting the MBL pathway is narsoplimab (OMS721), currently in phase II clinical trial for MN and phase III trial for IgA nephropathy (NCT02682407). As knowledge of complements role in the pathogenesis of MN grows, so too will the targeted approach with anti-complement therapy.

## Conclusion

The landscape for treatment of membranous nephropathy has undergone significant change over the last 5 years. Despite the rarity of the disease, we now have a number of randomized trials to clarify the use of immunosuppressants in MN. Rituximab has been established as superior to cyclosporine, and is at least comparable to cyclophosphamide-glucocorticoid based regimens for moderate to high-risk patients. Reflected in the updated KDIGO guidelines, the cyclophosphamide-based regimens remain first line for very high-risk disease to prevent loss of kidney function or life-threatening complications of nephrotic syndrome. The future of therapies will be exploring anti- complement, BAFF, and plasma cell targeting therapies, among others. However, as the diagnosis of MN is becoming more refined, so too will our existing and future therapies, ultimately providing each patient with an individualized therapeutic regimen.

## Author contributions

CC contributed to conception and design of the review and wrote the first draft of the manuscript. MS, AD contributed to manuscript revision, drafting, read, and approved the submitted version.
